# The Role of Local Instabilities in Fluid Invasion into Permeable Media

**DOI:** 10.1038/s41598-017-00191-y

**Published:** 2017-03-27

**Authors:** Kamaljit Singh, Hagen Scholl, Martin Brinkmann, Marco Di Michiel, Mario Scheel, Stephan Herminghaus, Ralf Seemann

**Affiliations:** 1 Saarland University, Experimental Physics, D-66123 Saarbrücken, Germany; 2 Max Planck Institute for Dynamics and Self-Organization, D-37077 Göttingen, Germany; 3 ESRF, The European Synchrotron, 71 avenue des Martyrs, 38000 Grenoble, France; 4 Imperial College London, SW7 2AZ London, UK; 5 Synchrotron Soleil, L’Orme des Merisiers, Saint-Aubin, 99190 Gif-sur-Yvette, France

## Abstract

Wettability is an important factor which controls the displacement of immiscible fluids in permeable media, with far reaching implications for storage of CO_2_ in deep saline aquifers, fuel cells, oil recovery, and for the remediation of oil contaminated soils. Considering the paradigmatic case of random piles of spherical beads, fluid front morphologies emerging during slow immiscible displacement are investigated in real time by X-ray micro–tomography and quantitatively compared with model predictions. Controlled by the wettability of the bead matrix two distinct displacement patterns are found. A compact front morphology emerges if the invading fluid wets the beads while a fingered morphology is found for non–wetting invading fluids, causing the residual amount of defending fluid to differ by one order of magnitude. The corresponding crossover between these two regimes in terms of the advancing contact angle is governed by an interplay of wettability and pore geometry and can be predicted on the basis of a purely quasi–static consideration of local instabilities that control the progression of the invading interface.

## Introduction

Immiscible displacement of one fluid by another within the pore space of a random permeable medium, also called fluid invasion, is of utmost importance in many settings in nature and technology, such as the penetration of soils by water or oil spills, storage of CO_2_ in deep saline aquifers, the production of crude oil from porous rock, the injection of fluid in filter cakes, and many more. Despite its enormous importance and a substantial body of previous research^1–10^, there is as yet no conclusive understanding of this process at pore–scale^11–25^.

The residual saturation with defending fluid during slow immiscible displacement is known to be influenced by the wettability of the pore walls^26, 27^. Experiments using dense random piles of spherical particles, the widely used paradigmatic model system^1–3, 8, 19–24^, have revealed that if the invading fluid wets the solid completely (vanishing contact angle, *θ* ≈ 0°), the front stays rather smooth and expels almost all of the defending phase, while a non-wetting front (*θ* ≈ 180°) acquires a ramified morphology, leading to a large residual fluid saturation^22–25^.

A similar cross–over has been found in a numerical model by Cieplak and Robbins^28, 29^, who considered a two-dimensional ‘fluid’ front invading an arrangement of circular disks. The centers of the disks were placed on a regular lattice, but their radii were varied in a random fashion in order to mimic the random nature of a porous medium. The sizes of the disks were such that neighboring disks were not in contact. The invading liquid front consisted of circular arcs of equal curvature, extending between disk perimeters and intersecting the latter at a prescribed angle, *θ*. The numerical calculations showed that if *θ* was small, the front remained rather smooth, while a large *θ* resulted in a very ramified front, in qualitative agreement with recent experiments in quasi–two–dimensional Hele-Shaw cells^30, 31^ as well as full scale^30^ and coarse-grained^32^ fluid dynamics simulations.

A qualitative explanation of this finding can be given as follows. The porous solid matrix divides the invading front into a large number of intruding fluid menisci, each of which has a certain Laplace pressure, *p*_*L*_ = 2*γκ*, where *γ* is the surface tension of the fluid–fluid interface, and *κ* its mean curvature. During the progression of the meniscus, the Laplace pressure varies as a consequence of the complex geometry of the medium. If *p*_*L*_ decreases with progressing fluid, the meniscus will burst into the next pore in an accelerated manner. This instability was originally described in a seminal work by Haines^2^ and is henceforth referred to as ‘Haines jump’ in the remainder of this paper. For the mentioned geometry of circular disks, it takes only elementary geometry to show that such jumps are quite unlikely if *θ* is small, but become very probable if the contact angle is large^28, 29^. In the numerical calculations performed for many different values of *θ*, it was generally found that whenever Haines jumps were occurring frequently, a ramified fluid front was observed in two dimensions. The frequent occurrence of Haines jumps, however, is tantamount to a reduction of cooperative pore filling events where the menisci of at least two throats are involved. As *θ* was gradually decreased, the frequency of Haines jumps displays a pronounced decrease while approaching a critical contact angle where the morphology of the front changed from rough to smooth^28, 29^. We shall call this the Cieplak–Robbins cross–over.

So far, such a steep wettability controlled cross–over of fluid displacement patterns in a fully three–dimensional porous matrix was found neither in experiments nor in full–scale fluid dynamic simulations. This is because a systematic variation of stable and reliable contact angles is hard to achieve in experiments. Fluid dynamics simulations that allow to study partial wetting conditions are computationally expensive because they demand a high spatial resolution close to moving three phase contact lines. Thus, it is not clear whether the pronounced difference of front morphologies that were found in a regular two–dimensional lattice of circular discs by Cieplak & Robbins^28, 29^ also exist in three dimensions. The fluid–fluid interface of the invading phase in the two–dimensional model of Cieplak and Robbins is a collection of well separated, circular menisci. Considering random piles of spherical beads as a model system in three dimensions, each bead has only a few point contacts with its neighbors in a stable mechanical equilibrium, and one generically encounters continuous interfaces between the fluid phases in the pore space. In view of the potentially strong cross–talk between interfacial equilibrium shapes in adjacent pores, it appears rather questionable that the highly idealized two–dimensional model of Cieplak and Robbins can be applied to a ‘real’ three–dimensional porous media consisting of a dense bead pack.

In fact, we show by *in situ* X–ray micro-tomography that the interface of a non-wetting fluid invading a random bead pile prefers to penetrate through larger throats while it develops a ramified morphology. A wetting fluid invading a random bead pile obeys no such preference in its flow path and the emerging front morphology remains compact. These observations strongly support the assumption that the invasion process is indeed quasi–static and dominated by non–cooperative interfacial instabilities for high contact angles^5, 6^. Using simple geometric arguments and the local geometry of the bead pack, we are able to compute the contact angle at the cross-over between the two displacement regimes where either cooperative or non-cooperative pore filling events dominate the local front advance. Not only the precise location of the cross–over, but also the relative frequency of non–cooperative Haines jumps as a function of the contact angle can be employed to predict the residual saturation of the defending phase. A strong correlation between the experimentally determined residual saturation and the relative frequency of Haines jumps corroborates once more that pore–scale processes during slow invasion are fully captured in the quasi–static picture put forward in this article.

## Experiments

Dense piles of spheres are employed as three–dimensional model porous media, with controlled surface contact angle *θ* with respect to the invading phase. The interstitial space of the samples was first filled with an oil (defending fluid), which was then expelled by an invading aqueous phase. The morphology of the fluid fronts and the residual saturation with the defending fluid were observed during invasion by X–ray micro-tomography. Volume flux controlled experiments were conduced at an average front velocity of *U* ≈ 3 *μ*m/s. Employing the dynamic viscosity *η* and density *ρ* of the invading aqueous phase, as well as the interfacial tension *γ* between the fluid phases, this velocity corresponds to a capillary number Ca = *ηU*/*γ* = 1.6 × 10^−7^, and to a Reynolds number of ${\rm{Re}}=\rho U\langle D\rangle /\eta =1.2\times {10}^{-3}$ based on the average bead diameter $\langle D\rangle$. Working at such low volume fluxes the fluid distribution could be imaged *in situ*, without any restrictions from the data acquisition rate. It was carefully checked that no systematic change, neither in the front morphology nor in the residual saturation of the pore space with the defending fluid, occurred when the displacement velocity was varied even by more than one order of magnitude. Buoyancy effects in the evolution of the fluid interfaces can be ruled out by the very small Bond number ${\rm{Bo}}=g{\rm{\Delta }}\rho {\langle D\rangle }^{2}/\gamma =0.0025$ where Δ*ρ* denotes the density difference between the invading and defending phase, and *g* the acceleration of gravity. Given the small value of the Bond number, being two orders of magnitude smaller than unity, capillary pressure variations caused by hydrostatic pressure gradients across the imaged field of view can be safely neglected^33^. This is supported by repeats of the experiments with the sample cell turned upside down and thus with inverted flow direction that gave no noticeable changes of the displacement patterns. We finally note that the viscosities of the aqueous and the oil phase were similar, thus ruling out a Saffmann-Taylor–type instability as responsible for shaping the fluid front. Moreover, no changes in the morphology of the displacement patterns were observed when the viscosity of the oil phase was increased by one order of magnitude.

**Figure 1:**
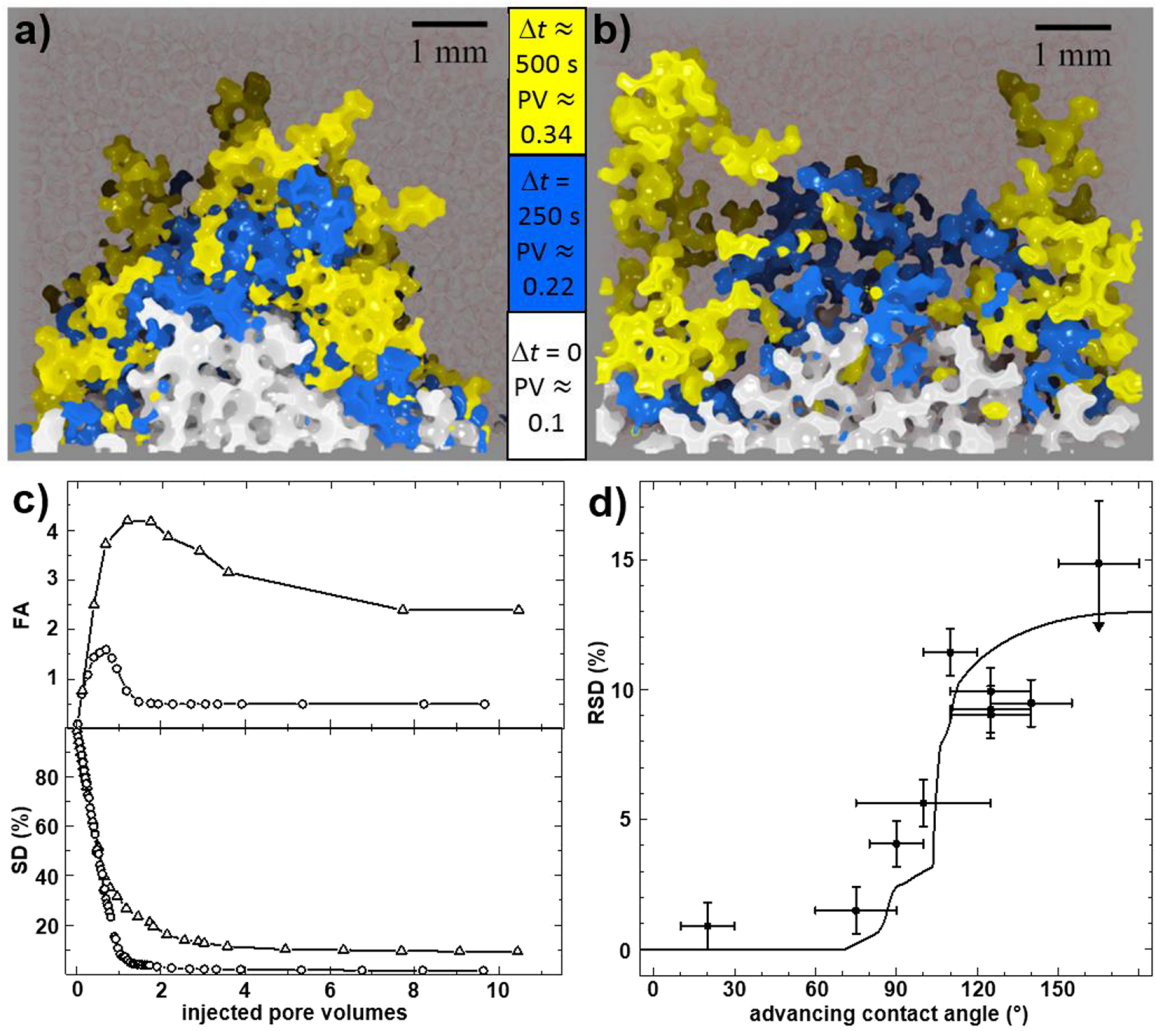
Evolution of an invading liquid front for different contact angles in random bead packs. (**a**,**b**) Water morphologies invading an oil filled random pile of spherical beads, as revealed by X-ray micro-tomography. Only a slice with a depth of four bead diameters is shown and beads are rendered semi–transparent (reddish hue) for clarity. Different colors of the front correspond to different times *t* and injected pore volumes (PV). (**a**) basalt beads, *θ* = (75 ± 15)°, (**b**) glass beads *θ* = (125 ± 15)°. (**c**) *top*: Interfacial area of the invading fluid an﻿d *﻿bottom*: Sa﻿turation with defending phase﻿ as a func﻿tion of ﻿the injected pore volume of the invading ﻿phase (given in units of the total observed pore volume in the imaged field of view) ﻿for basalt beads (open circles), glass be﻿ads (open triangles). The interfacial area was determined as the sum of the fluid–fluid and the fluid–solid interface excluding the completely immersed beads and ganglia of the defending phase and normalized with the sample cross section﻿. The finite plateau values for large injected volumes reflect liquid structures extending across the field of view. (**d**) Residual saturations as a function of contact angle *θ*. The arrow at the rightmost datum point indicates that at these large contact angles, relaxation times exceeded the duration of the experiment and we could not reach the point where the defending fluid depercolates. Accordingly a lower value of the residual saturation is expected.

Two examples for a volume flux controlled fluid invasion into bead packs with uniform, but different contact angles are shown in Fig. 1a and b. If the contact angle *θ* of the invading phase is well below 90°, the front is observed to be smooth, with the total interfacial area of the invading fluid remaining less than two times the sample cross–section throughout, c.f. the circle symbols in Fig. 1c. The invading fluid fills almost all of the pores, and the saturation of the medium with the defending fluid quickly drops to a very small plateau value $\lesssim$ 1% indicating the residual saturation (Fig. 1c bottom). If the contact angle *θ* of the invading phase is larger than *θ* ≈ 110° (c.f. Fig. 1b), a ramified front emerges the interfacial area of which attains more than four times the sample cross section (Fig. 1c, triangles). This fingered structure of the front results in a dramatically increased residual saturation of 10% despite the highly connected and regular pore space. Plotting the obtained residual saturation as a function of the advancing contact angle, *θ* (Fig. 1d), we clearly observe a sharp rise of the residual saturation above a critical angle of a little less than 90°.

**Figure 2:**
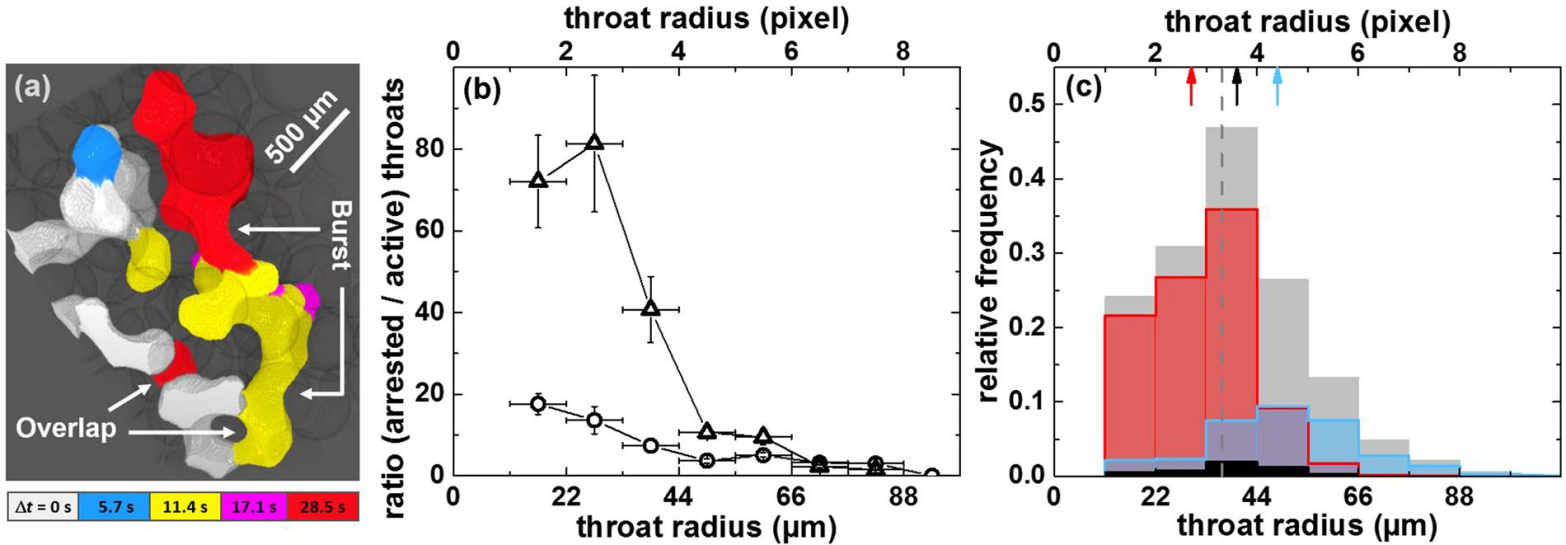
Pore-filling events and throat size analysis during water injection. (**a**) Three-dimensional volume rendering of five time steps during fluid invasion into glass beads (semi–transparent), *θ* ≈ 125°. These images were recorded with a time interval of 5.7 s using the on-board memory of the camera. The aqueous phase was injected at an average front velocity of 3 *μ*m/s. The progressing fluid front is shown in white, blue, yellow, magenta and red. (**b**,**c**): Analysis of the large scale experiments shown in Fig. 1 recorded with a time interval of 250 s. Throats and menisci determining the position of the fluid front are identified in a first time step. The fraction of those menisci and throats which still determine the position of the fluid front in a consecutive time step are called ‘arrested throats’ while the fraction of those throats which got filled are termed ‘active throats’. Data are averaged for ten consecutive time steps when the invasion front was already fully developed but not yet exceeded the imaged volume (0.1 $\lesssim$ PV $\lesssim$ 0.5). (**b**) Fraction of ‘arrested throats’ divided by ‘active throats’ as a function of throat size for glass (triangles) and basalt beads (circles) (*θ* ≈ 75°). The errors in *x*- and *y*-direction denote the bin size and the mean value, respectively. (**c**) Histograms of arrested throats (red), active throats (black), and throats that became filled within the respective time interval and which were not present at the fluid front neither in the first nor in the second time step (blue), normalized by the total number of throats at the fluid front. The throat size distribution of the entire bead pack (gray) is shown at a different scale for better visibility. The corresponding mean values of the distributions are indicated by arrows, and, for the entire bead pack, by the gray dashed line.

To further explore the evolution of the interface morphology, we analyzed the progression of the invading fluid at the level of individual pores and throats. A typical result observed for large contact angles is shown in Fig. 2a. The invading non-wetting fluid intrudes locally only through a single active ‘finger’ which sometimes advances over several pore diameters. This mode of local front advance is expected for non–cooperative Haines jumps under quasi–static conditions. In accordance with the invasion percolation models^5, 6^, the interface of the invading fluid progresses only through the largest throats as these need the smallest Laplace pressure to be traversed. At the smaller throats, the interface remains arrested in the respective step. Cooperative effects involving several menisci are expected to be less size sensitive, and could not be observed in those short sequences. The selectivity of the throat sizes can be illustrated for longer sequences of interfacial advances by considering the ratio of the number of arrested menisci, divided by the number of throats filled in the same time step, c.f. Fig. 2b. For the sample with large contact angle (glass, *θ* ≈ 125°), we clearly see that the interface of the invading fluid is selectively arrested in the smaller throats, and advances preferentially through the larger ones. For small contact angles (basalt, *θ* ≈ 75°), this selectivity is absent, as the corresponding curve shows. This observation is in line with the expected presence of cooperative effects and explains the smooth fronts observed for small contact angles.

Returning to the long invading ‘finger’ structure from Fig. 2a, we need to investigate whether inertial (or other dynamic) effects must be invoked to explain such events^34^, or whether they conform to the preconditions of a quasi–static description of the invasion process. The quasi–static approach is justified whenever the interface stays close to a stable mechanical equilibrium for most of the time. However, in the course of an interfacial instability, i.e. after mechanical stability has been lost, the motion of the fluids in the quasi–static picture is governed by a fully over–damped dynamics. Under this condition, the fluid interfaces move exclusively into the downhill direction of the free energy landscape and evolve into the next local energy minimum.

An estimate for the local interfacial velocity during a pore–filling event can be obtained from the sequence of fast X–ray tomography images presented in Fig. 2a. If we assume a continuous advance of the liquid fingers, the corresponding local velocity and Reynolds number with respect to the average bead diameter is *U* ≈ 0.5 mm/s and Re ≈ 0.5, respectively. However, the duration of a filling event can be shorter leading to even larger local velocities^34–36^.

Even though we cannot rule out inertial effects completely, a statistical analysis of the filling events observed within the entire sample volume demonstrates that inertia does not govern the progression of the front. Both the average radius of throats invaded in a primary step and the average radius of throats invaded in a secondary step, which could not time resolved, are larger than the average throat radius, Fig. 2c. Importantly, the average radius of throats invaded in a secondary step is systematically larger than the average radius of throats that have been invaded in a primary step. This selectivity is expected for liquid rearrangements that follow a fully overdamped interfacial dynamics, as demanded for a quasi–static invasion percolation process^5, 6^.

## Theory

**Figure 3:**
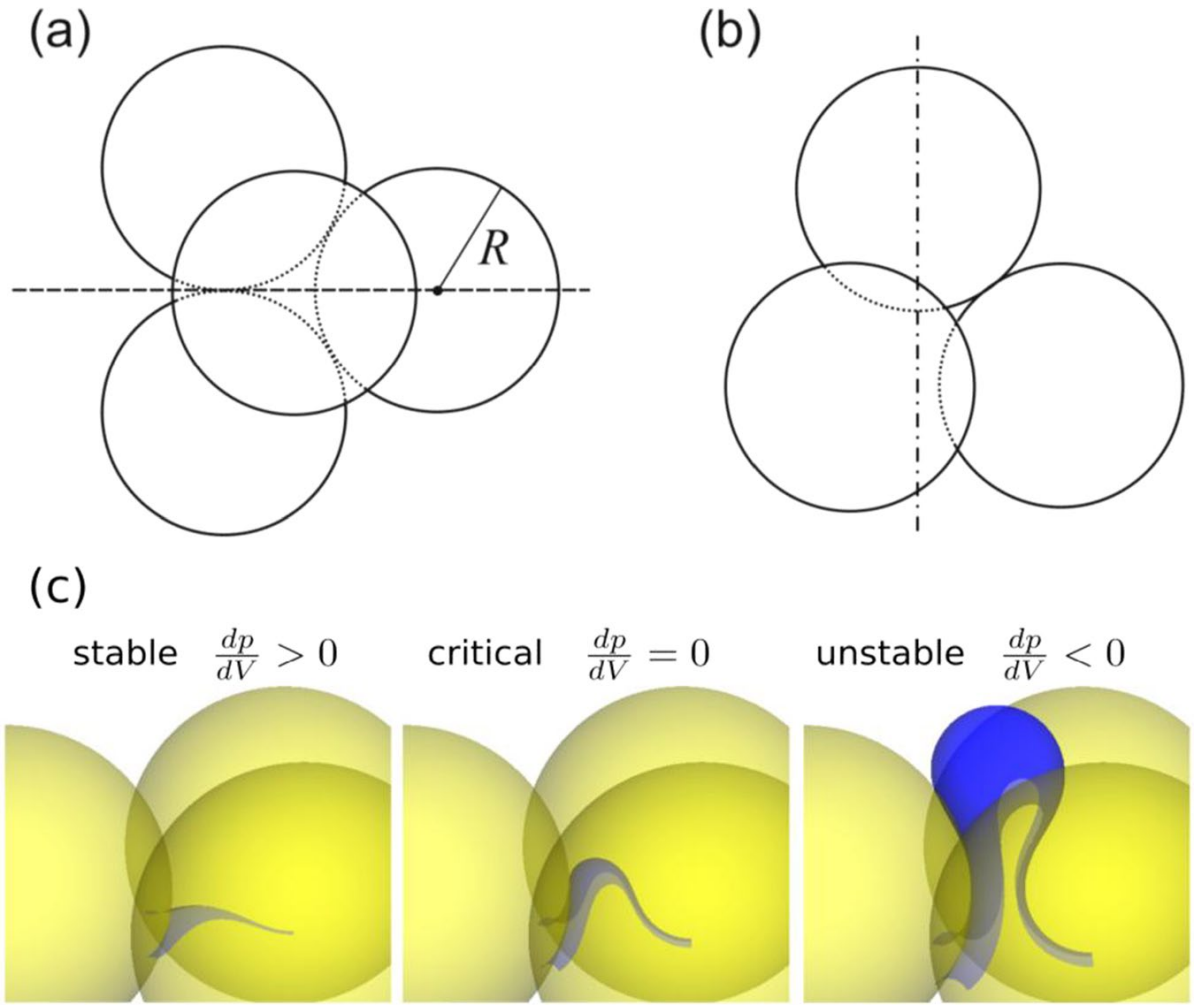
Sketch of a tetrahedral arrangement of spheres, as it frequently appears in random piles of spherical particles. (**a**) Top view and (**b**) Side view: The dash-dotted line indicates one of the four symmetry axes of the tetrahedron for rotations by 120°. The lower left circle represents two spheres one of which eclipses the other. The fluid interface invades from below. (**c**) Sequence of numerically computed constant mean curvature surfaces in a perfect triangular throat for a contact angle of *θ* = 170°. The image in the middle shows a critical meniscus, i.e. at the point of its largest Laplace pressure.

In order to predict the contact angles at which Haines jumps are to be expected in three dimensions, we consider the geometry of pore invasion in a (random) pile of spheres in a quasi–static setting. Note that in three-dimensions, a densely packed pile of spheres is both porous and permeable, as opposed to dense two–dimensional packing of disks. As a most simple model of a pore, we start by considering four spheres of equal size (radius *R*) in mutual contact, which we call an ideal tetrahedral pore, c.f. Fig. 3a and b. In the ideal tetrahedral pore, the contact points on each sphere enclose an angle of *α* = 60° with respect to its center.

Let us assume that the fluid front invades a tetrahedral pore from below in the side view sketch in Fig. 3b, such that the dash-dotted line is normal to the front. As the fluid invades the nook between the three bottom spheres, its shape may be well approximated by a sphere the center of which lies on the dash-dotted line. This can be appreciated by remarking four facts. First, the invading front must be a constant mean curvature surface due to pressure balance. Second, a possible solution for a constant mean curvature surface intersecting three given spheres at a prescribed contact angle is a sphere. Third, as the two principal curvatures of any surface are perpendicular to each other, the leading term in any deviation from a sphere must have four–fold symmetry. The invaded nook, however, has a threefold symmetry and hence can perturb the bulge only to higher order. Fourth, the bulge invading the nook under consideration will be only weakly perturbed by the bulges invading the neighboring nooks, because the contact line will be strongly pinned close to the contact points of adjacent spheres. This pinning results from the small distance of the solid surfaces in the vicinity of the contact points, because this would give rise to large changes of curvature if the contact line were moved appreciably. Hence any potential cross–talk between neighboring bulges is impeded by the proximity of the contact points. Away from the contact points, the bulge is then very well approximated by a sphere, and only weakly perturbed by the geometry of neighboring parts of the front. For illustration, Fig. 3c shows liquid bulges obtained from numerical energy minimizations that invade a hexagonal array of spheres in mutual contact. The obtained liquid bulge can be in fact approximated by a sphere that is located in the nooks. Similar morphologies have been described before for various contact angles^37^.

If the contact angle *θ* between the fluid–fluid interface and the solid surface is given, it is straightforward to calculate the radius of curvature, and hence the Laplace pressure, *p*_*L*_, of the (assumed spherical) bulge as a function of the position of its center along that line. It is obvious that if the top sphere were absent, *p*_*L*_ would at some point start to decrease. If this happens before the bulge touches the surface of the top sphere, a Haines jump occurs. This sudden decrease of the Laplace pressure during invasion of a non–wetting fluid into a pore was first described by Haines as a *per saltem* motion of a fluid–fluid interface^1^.

**Figure 4:**
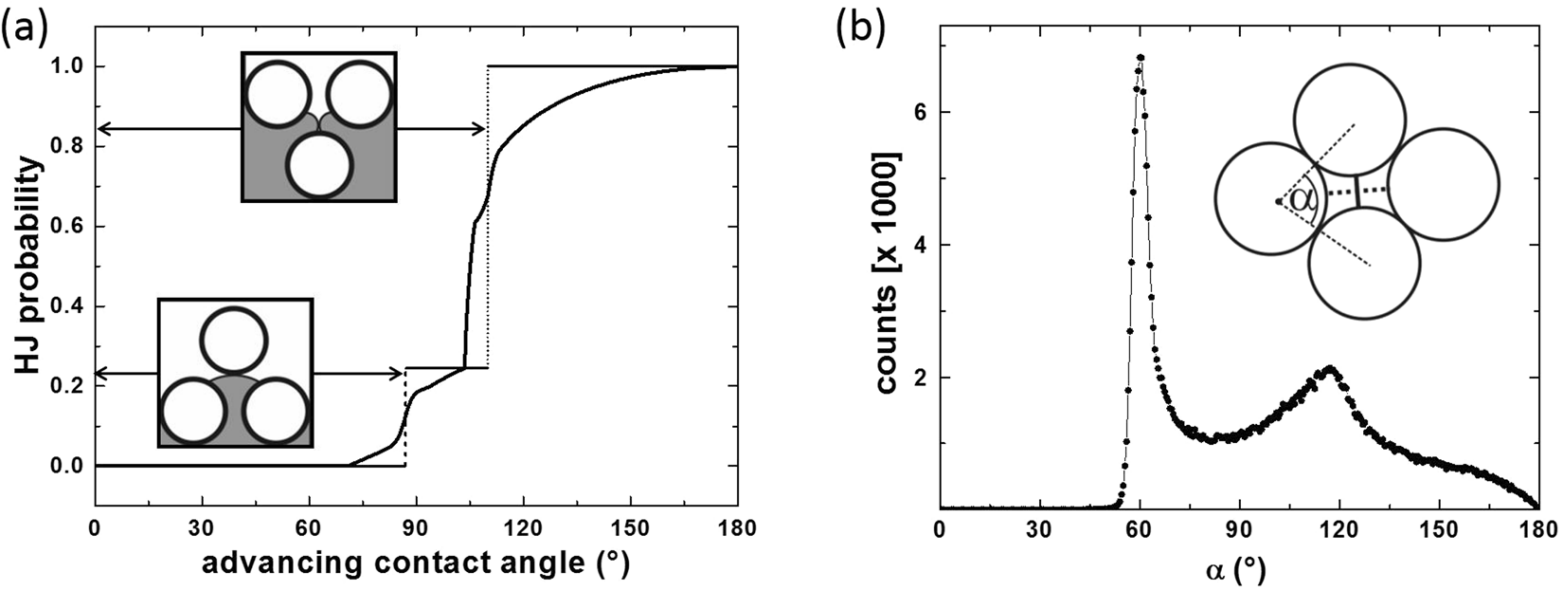
Probability that a pore is being filled by a Haines jump. (**a**) Theoretically derived probabilities as a function of the advancing contact angle *θ* of the invading phase. The result for ideal tetrahedron pores is shown by the dashed and dotted step function. The result for more realistic tetrahedron pores with one opening is shown by the solid line. (**b**) Distribution of mutual angles between spheres in a random packing, as determined experimentally by X–ray micro-tomography. The strongly dominant peak at *α* = 60°, together with its ‘mirror image’ at 120°, corroborates that tetrahedral pore with spheres in mutual contact prevail.

The condition for a Haines jump in a tetrahedral pore is that $\theta \gtrsim {87}^{\circ }$, which is elementary to compute from the geometry of the tetrahedral pore (see Supplementary Information). In Fig. 4a this is indicated by the vertical dashed line. A Haines jump can be precluded not only by the bulge reaching the opposite pore wall, but also if it touches a second bulge entering the pore through a neighboring throat. This would be needed to be considered if the interface in the sketch Fig. 3a were advancing into the pore from above instead of from below, and could be similarly calculated from simple geometric considerations. The result is a critical angle of *θ* ≈ 110°, which is displayed in Fig. 4a as the vertical dotted line. Both values are remarkably close to the region where the sharp rise in the residual saturation of the defending fluid is observed, cf. Fig. 1a.

We can compute the probability for Haines jumps by considering the statistical weights for the various orientations of the pore with respect to the invading front. Each pore has, by its very definition, four throats through which it can be entered. We know for sure that the pore under consideration is still empty, and it is located at the front. Hence neither all four throats are being invaded at once (then the pore would be already immersed in the invading fluid), nor do they all remain empty (then the pore would still be ahead of the front). Consequently, we have to distinguish three cases: either the pore is invaded by one bulge in which case the Haines jump may be precluded by touching the opposite pore wall, or by two or three at the same time. In the two latter cases, the Haines jump is precluded by two or three bulges coalescing. As the pore under consideration is located at the invading front, we may assume that the probability that a neighboring pore is already filled is 1/2 (for each neighboring pore separately). Hence if the orientation of the pore with respect to the invading front is random, the statistical weight of the pore being filled by one bulge invading from the ‘bottom’ or by three bulges from ‘top’ (cf. Fig. 3b) is 1/4 each, while for the second possibility it is 1/2. Respecting these statistical weights we arrive at a stepped function for the frequency of Haines jumps during the invasion, which is indicated in Fig. 4a.

Now we can go one step further and consider a more realistic pore model. In a real random pile of spheres, not all spheres of a tetrahedral pore are in direct contact, thus enclosing a smooth distribution of angles around *α* = 60° with respect to the center of the respective sphere (see inset of Fig. 4b). To discuss the influence of non–ideal pores, we adopt a (leading order) approximation assuming that among four neighboring spheres enclosing what we call a pore, there is at most one open contact (corresponding to *α* > 60°). For this geometry, the evaluation of the critical angles for occurrence (or preclusion) of Haines jumps is more tedious, but still analytically straightforward (see Supplementary Information). Instead of a single critical angle, we now obtain, for every orientation of the pore with respect to the invading front, a distribution of critical contact angles which derives directly from the distribution of *α* depicted in the inset of Fig. 4b.

As a final step, we take the distributions of critical contact angles precluding Haines jumps for the three possible orientations of the pore, multiply them by the corresponding statistical weights, and add them all up. By virtue of this calculation, we can derive the probability of Haines jumps to occur in a random pile of spheres as a function of the contact angle. The result is plotted as the solid curve in Fig. 4a. We see that the transitions acquire some inhomogeneous broadening due to the randomness of the pile, but qualitatively the steep increase remains largely unaltered.

## Discussion

**Figure 5:**
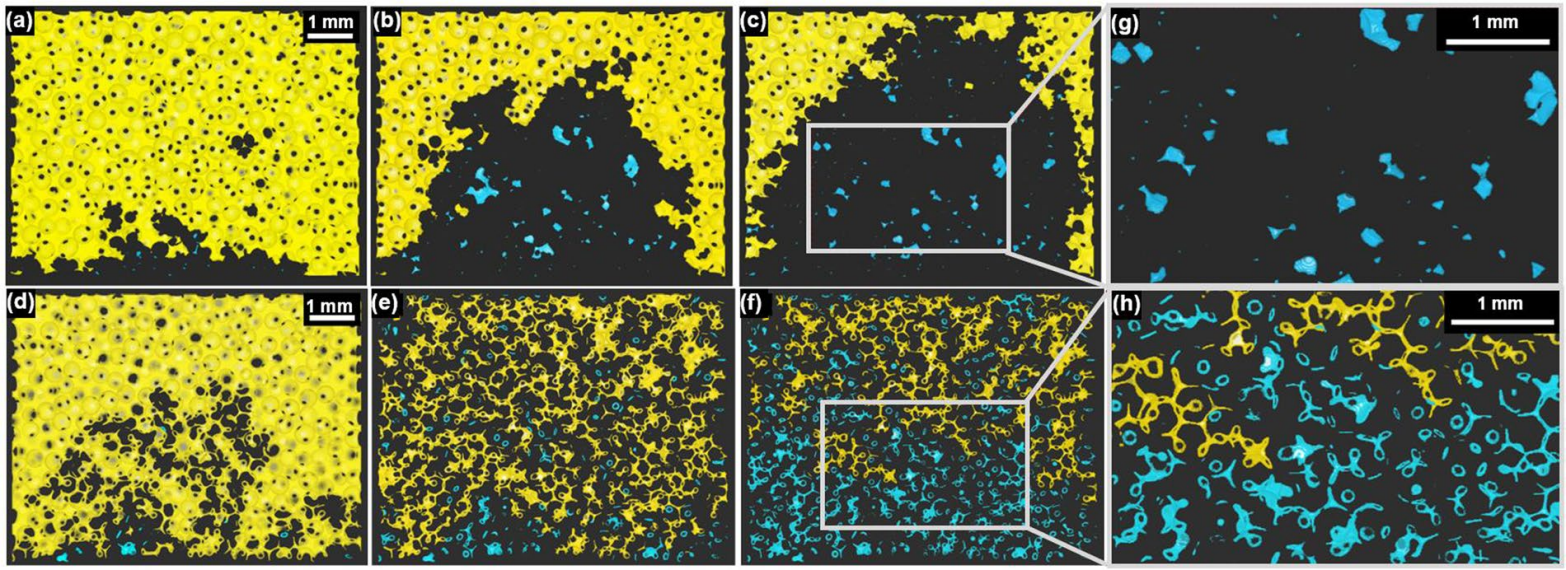
Percolated and disconnected oil morphologies during water injection. Time series of vertical cross-sections of one bead layer thickness. The percolated oil is shown in yellow and the disconnected oil ganglia are shown in blue. Beads and the defending phase are rendered transparent. (**a**), (**b**) and (**c**) represent time series of basalt beads with *θ* ≈ 75° after 0.1, 0.4 and 0.7 injected pore volumes (PV). (**d**), (**e**) and (**f**) represent time series of glass beads with *θ* ≈ 125° after 0.3, 2.9 and 10.4 injected PV. Zoomed section for basalt (g) and glass beads (h) after 0.7 and 10.4 injected PV, respectively.

What is still missing to complete the picture of fluid invasion at different wetting conditions is a connection of the probability of Haines jumps to what is presumably the most important parameter in fluid invasion, the residual saturation. Recall that significant residual saturation is found at contact angles above the cross–over. This is, in particular, at angles $\theta \gtrsim {90}^{\circ }$ for the invading phase. Consequently, the defending phase makes a contact angle $\theta \lesssim {90}^{\circ }$ with the surface of the beads and accumulates as pendular rings at the contact points between adjacent grains. Hence when the invading fluid passes a throat, the defending fluid acquires a morphology reminiscent of a wetting liquid in a granular pile. This type of morphology is known to consist of characteristic ganglia, which are shaped like single capillary bridges, capillary bridge trimers, pentamers, and larger agglomerates of this type of structures^34^. These morphologies can be seen in the tomography images shown in Fig. 5 (bottom row) for glass beads with a contact angle of *θ* ≈ 125°, but are absent in corresponding packings of basalt beads with *θ* ≈ 75°, c.f. Fig. 5 (top row). When inspecting both the percolated (yellow) and the ruptured fragments (blue) of the defending phase in the pile of basalt beads, where the liquid has no preferred flow path and the invading front was found to be smooth, the residual oil remains in irregular shaped individual liquid morphologies filling, e.g., a tetrahedral pore. In contrast, for glass beads, where the invading fluid penetrates preferentially through the largest throats and develops a ramified front, we can clearly identify the above mentioned building principle of the remaining morphologies of the defending phase. In the regime dominated by Haines jumps (ramified interface), an interconnected network of invading and defending phase is formed at the late stage of fluid invasion that extends over a substantial distance (bottom row of Fig. 5). Note that these types of liquid morphologies also appear in piles of irregularly shaped sand grains^38^, such that our reasoning is not restricted to the considered spherical bead packs but should be more general provided the beads have convex contacts.

**Figure 6:**
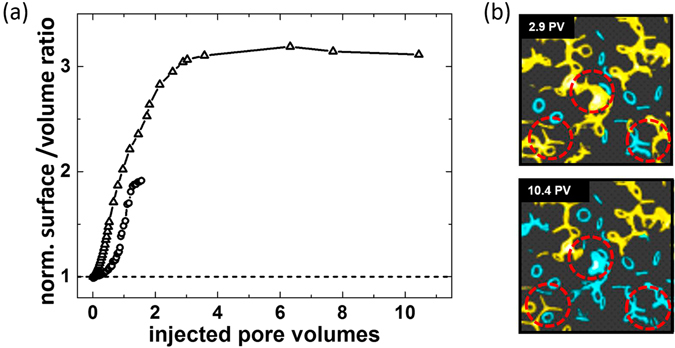
Surface–to–volume ratio of the percolated oil phase during a water flood. (**a**) Symbols display the ratio *a*/*a*_0_ of the surface–to–volume ratio *a* of the defending phase to the value *a*_0_ for a random close pack of beads initially fully saturated with the defending phase. Shown are data for basalt (circles) and glass (triangles) with contact angles *θ* ≈ 75° and *θ* ≈ 125°, respectively, as a function of the injected pore volume. (**b**) Three-dimensional volume rendering of the defending phase during aqueous phase injection into glass beads after 2.9 and 10.4 injected pore volumes. Areas where the disconnection of ganglia during drainage of defending phase are clearly visible are indicated by circles.

In order to quantify the temporal development of the interfacial morphology of the defending phase during invasion, we consider the evolution of the ratio *a* = *A*/*V* of surface area *A* to volume *V* of the defending phase. Figure 6a displays the surface–to–volume ratio *a* of the percolated subset of the defending phase normalized by the value *a*_0_ for a random close pack of beads fully saturated with the defending phase. For the smaller contact angle of the invading phase (basalt *θ* ≈ 75°) the surface–to–volume ratio *a* remains small at a maximum value corresponding to about twice the surface–to–volume ratio *a*_0_ as expected for compact morphologies in a random close packing of beads^38^. In the case of large contact angle (glass *θ* ≈ 125°, ramified morphology), we find an asymptotic value of *a*_∞_ ≈ 3.3*a*_0_ of the surface–to–volume ratio for the percolated oil phase after injecting several pore volumes of the non–wetting invading phase (PV $\gtrsim$ 5). This value of the surface–to-volume ratio is in fact close to the value reported for the liquid clusters of a wetting fluid in granular piles^38^. The large ratio *a*_∞_/*a*_0_ is a consequence of the particular invasion process by which a non–wetting fluid displaces a wetting fluid from a permeable solid. During the fluid invasion at large contact angles, a percolated network of oil emerges, intertwined with a dense network of fingers of the invading aqueous phase that have formed due to the frequent occurrence of Haines jumps. This network of the defending oil phase does not change qualitatively but quantitatively upon further liquid injection. The network of defending phase is drained in the direction of flow, reducing its volume in the field of view, but hardly its surface area, finally leading to the asymptotic plateau value of *a*_∞_ ≈ 3.3*a*_0_, close to the value of the (de−) percolation threshold. Upon further liquid injection, small liquid ganglia of the defending phase detach from the percolated network and remain behind the front (Fig. 6b). The residual ganglia are mostly composed of a small number of connected capillary bridges.

The residual distribution of oil ganglia for invading phases with large contact angle *θ* is obviously a result of a de–percolation process of the defending phase and can thus be compared to the distribution of a wetting liquid in a bead pile at the percolation threshold. For bead packs wetted with a small contact angle liquid, it has been shown that percolation of the equilibrium liquid morphologies sets in at a liquid content just above 5% with respect to the total sample volume^38, 39^, i.e., at about 13% of the pore volume considered here. As long as there is more defending fluid in the medium than 13%, the invading fluid can expel it, with the percolated fluid structure serving as drainage channels^40^. This process comes to an end as soon as the residual saturation density (RSD) drops below 13% of the pore volume (PV). As a consequence, we expect that whenever the front is ramified the maximum RSD is about 13% of the PV. Because ramified fronts and the corresponding regular morphologies of the residual defending phase only emerge globally or locally for large contact angles where Haines jumps occur, it suggests itself to link the residual saturation of the defending fluid to the probability of Haines jumps. For a rough estimate of the residual saturation as a function of the contact angle, we multiply the probability of Haines jumps from Fig. 4a by 13%, displayed as the solid curve in Fig. 1a. The agreement with the experimental values is remarkable.

## Conclusions

In conclusion, our experiments clearly demonstrate that smooth fronts emerge upon invasion of a wetting fluid into a random pack of spherical beads, while ramified fronts emerge when non–wetting liquids are injected. Ramified fronts lead to residual saturations of the defending phase that are about one order of magnitude above the values found for smooth fronts. The build up of ramified fronts is the result of the selectivity of the flow path following the largest throats that progresses mainly by Haines jumps following the largest throats. During each Haines jump the local Laplace pressure of the respective liquid meniscus suddenly drops upon progression causing the fluid to invade the neighboring pore in an accelerated manner. As a result of these fluid ‘bursts’, an intertwined network of the invading and the defending phase emerges. The network of the defending phase slowly drains during further invasion and forms characteristic morphologies upon de–percolation which remain behind the ramified front.

Important features of fluid invasion into a three–dimensional porous matrix observed in the presented X–ray tomography data can be well understood on the basis of a strikingly simple, analytically tractable geometrical consideration, invoking only quasi–static concepts. The residual saturation with defending fluid can be predicted quantitatively. The overall behavior is found to be governed by a cross–over in the front morphology which occurs at a contact angle *θ* ≈ 100° above which Haines jumps are the dominating local instability. Given the known generality of liquid morphologies in wet granular piles^38^, our results are expected to remain valid to some extend for unconsolidated and consolidated granular piles consisting of a large class of convex grains. By establishing the probability of Haines jumps as the main mechanism which governs fluid invasion, our results also clarify why pore–network models are often found to have very limited predictive power for quasi-static fluid invasion, even if throat and pore size distributions are well in line with the sample characteristics^41^. It is the assumption of constant cross–section of the throats inherent in these models, which inhibits any non–monotone variation of Laplace pressure with front progression. Hence such simple pore network models have a blind spot which precisely eclipses the relevant mechanism in the quasi-stationary case. Our results may be instrumental for adopting pore network model appropriately to the porous media they are meant to model.

## Materials and Methods

### Porous media and sample cell

Permeable media employed in our experiments consist of dense random packs of glass or basalt beads (Whitehouse Scientific Ltd., UK) with sieve fraction of (355–425) *μ*m. The average bead diameter was determined by X–ray tomography to (383 ± 25) *μ*m and (414 ± 9) *μ*m, respectively. The basalt beads were cleaned with ethanol resulting in a water contact angle in a surrounding dodecane phase of *θ* = (75 ± 15)°. To achieve very small contact angles of (20 ± 10)° with the aqueous phase, glass beads were sonicated with organic solvents (ethanol, acetone, toluene), cleaned with piranha etch (50:50 mixture of H_2_O_2_ and H_2_SO_4_) and carefully rinsed with hot Millipore water^*TM*^. For very large contact angles of *θ* = (165 ± 15)° the thus cleaned glass beads were additionally coated with a self-assembled monolayer of octadecyl-trichlorosilane molecules^42^. Intermediate contact angles of (60–140)° were achieved by various combinations of beads, cleaning procedures (sonication only in water or ethanol) and different oils like dodecane or silicon oil (AK0.65, AK10), as summarized in Table 1.

**Table 1:** Summary of the (advancing) contact angles *θ* of the invading phase on the beads, surface tension *γ* between the invading and defending phase, and the viscosity ratios *η*_1_/*η*_2_ of invading and defending phase for different materials combinations used in this study.

liquid pair	bead material	*θ* [°]	*γ* [mN/m]	*η*_1_/*η*_2_
Air - water	glass	20 ± 10	70	0.02/1
Dodecane - water	basalt	75 ± 15	47 ± 1	1.4/1
AK0.65 - water	glass	90 ± 10	40 ± 1	3.3/1
Dodecane - water	glass	125 ± 15	47 ± 1	1.4/1
AK100 - water	glass	135 ± 15	42 ± 2	100/1
AK10 - water	glass	140 ± 15	42 ± 2	10/1
AK1000 - water	glass	140 ± 15	45 ± 1	1000/1
Dodecane - water	glass (OTS)	165 ± 15	47 ± 1	1.4/1

Ultrapure water doped with ZnI_2_ (Fischer Scientific, Germany) with a density of 1080 kg/m^3^ and a viscosity of *η* ≈ 1 mPa · s, was used as the invading phase. The ZnI_2_ dopant guarantees a sufficient X-ray attenuation to distinguish the aqueous phase from other phases present in the system whereas the viscosity is hardly affected^43^. A mixture of bromododecane - 8% by volume - in dodecane (both Sigma-Aldrich, Germany), in the text termed as ‘dodecane’, is used as the oil phase having a density of 761 kg/m^3^ and a viscosity of 1.383 mPa · s^44^. The bromododecane also increases the X–ray attenuation and allows to resolve potentially remaining or emerging gas bubbles. Dodecane was filtered three times through a column of aluminum oxide powder^45^ to remove impurities and to obtain a stable water-oil interfacial tension of *γ* = (46.7 ± 0.2) mN/m as determined by the pendant drop method. A similar purification or dopant was not required for the used silicon oils. The advancing contact angle at the beads’ surfaces, as given in Table 1, was measured by optical goniometry from beads dropped onto the oil/water interface.

The fluid invasion experiments were conducted in cylindrical polycarbonate cells (8 mm inner diameter, 58 mm height). The top and the bottom of the cell consist of cylindrical caps with a 4 mm and 1.2 mm diameter hole, respectively. A Whatman #40ashless filter paper was placed on the top of the base inlet of the cell to inject a homogeneous front at the start of the experiment. The reservoir in the top cap is sufficiently wide so that the fluid level in the reservoir is about constant during fluid invasion and the hydrostatic pressure only increases by replacing the lighter oil phase with the heavier aqueous phase. The inner side of the cell is additionally coated with cross-linked polydimethylsiloxane that swells in contact with the oil phase and prevents bypassing of the aqueous phase through the larger throats along the cell wall. The cell was first filled with the oil phase, and the beads were dropped in portions from the top and tapped between each layer to guarantee a random close packing. Then the top cap was pushed into the cell to firmly arrest the bead matrix. The resulting homogeneous bead packs have packing fractions of *ϕ* = 0.61 ± 0.01 and average throat radii of (37 ± 17) *μ*m, average throat length of (380 ± 130) *μ*m, and average pore radii of (70 ± 25) *μ*m. For all experiments the aqueous phase is injected into the bead matrix using home built computer controlled syringe pumps at constant volumetric flow rate of 61 nL/s leading to an average front velocity of 3 *μ*m/s.

### X-Ray tomography and image analysis

The fluid invasion was observed *in situ* during continuous flow by X–ray micro-tomography using the available setup at beamline ID15A of the European Synchrotron Radiation Facility in Grenoble, France. X–ray photon energies in our experiments ranged from 47 to 54 keV, providing the best results with respect to signal to noise ratio and X-ray dose/damage. The sample was placed on the rotation stage of the tomography setup and the aqueous phase was pumped from below towards the top. The fluid invasion was recorded within an image height of 6 mm close to the bottom of the sample cell. A series of absorption projections (radiographs) was collected by rotating the sample by 180°. An exposure time of about 0.35 ms was used to record each of the radiographs. The three–dimensional tomographic images (tomogram) were reconstructed from the radiographs using a filtered back projection algorithm. To minimize the X–ray dose while recording time series, X–ray radiations between two consecutive tomograms were blocked with a fast shutter and no visible X-ray damage was detected.

The voxel resolution was 11 *μ*m and typically 4000 radiographs were recorded during a total data acquisition time of 1.4 s. All radiographs belonging to one tomogram were downloaded from the CMOS camera (PCO Dimax) after acquisition and saved to a hard drive providing a nearly unlimited number of tomograms. The downloading time however, required an interval of at least 30 s between consecutive tomograms. To record tomograms at higher time resolution as acquired for Fig. 2a, 3000 radiographs per tomogram were recorded within 1.05 s and stored in the on–board memory of the camera allowing for short time intervals of 5.7 s between consecutive images at cost of a limited number of tomograms.

All reconstructed tomograms were processed for phase identification using the Mango software toolkit^46^, including edge preserving anisotropic diffusion filtering. In a first step, the bead phase recorded prior to an experiment was subtracted from the subsequently recorded time series. In a second step, the remaining two fluid phases during fluid invasion were segmented. The segmented data were used both for visualization purposes using Drishti volume rendering software^47^ and for the quantitative analysis.

## Electronic supplementary material

The Role of Local Instabilities in Fluid Invasion into Permeable Media

S1 Basalt bead pack

S2 Glass bead pack

S3 Fast-tomos
