# Supplementary material for: The Role of Local Instabilities in Fluid Invasion into Permeable Media
Source: Sci Rep. 2017 Mar 27;7:444. doi: 10.1038/s41598-017-00191-y (PMC5427855; doi:10.1038/s41598-017-00191-y)
Supplement: Supplementary file 1 — The Role of Local Instabilities in Fluid Invasion into Permeable Media [file 41598_2017_191_MOESM1_ESM.pdf]

The Role of Local Instabilities in Fluid Invasion into Permeable Media

Kamaljit Singh,[1,2,3,4] Hagen Scholl,[1,3] Martin Brinkmann,[1,3] Marco Di Michiel,[2] Mario Scheel,[2,5] Stephan Herminghaus,[3] and Ralf Seemann[1,3,*]

[1]*Saarland University, Experimental Physics, D-66123 Saarbrücken, Germany*
[2]*The European Synchrotron, 71 avenue des Martyrs, 38000 Grenoble, France*
[3]*Max Planck Institute for Dynamics and Self-Organization, D-37077 Göttingen, Germany*
[4]*Current Affiliation: Imperial College London,SW7 2AZ London, UK*
[5]*Current Affiliation: Société civile Synchrotron SOLEIL, BP 48, F-91192 Gif-sur-Yvette Cedex*

Contact Angle Dependent Transition of Fluid Invasion

Ideal tetrahedral pores

A tetrahedral arrangement of spheres enclosing a pore is sketched in Fig. 1. All grains are considered spherical, of equal size, and in mutual contact. This will be called an *ideal pore* below. We study the invasion of a liquid interface into the pore from different directions.

Single liquid bulge invading

Considering Fig. 1a as a top view, we discuss the invasion of a liquid front from below. At zero Laplace pressure, the invading interface can be just a horizontal plane, which is perpendicular to the vertical dash-dotted line in the side view, Fig. 1b. Its vertical position would arrange such as to accommodate the prescribed contact angle, θ, with the surfaces of the three spheres at the bottom. It is clear from the symmetry of the system that this angle would, as required, be constant along all three circular intersections of the plane surface with the spheres. If, for instance, the contact angle is $\pi/2$, the liquid would wet exactly the lower half of each of the three spheres at the bottom.

If the Laplace pressure, p_L, is finite, the liquid surface will acquire a more general shape. It will have constant mean curvature and intersect each of the three bottom spheres at an angle θ. A simple and obvious solution is a spherical surface with radius $r = 2\gamma/p_L$, the center of which lies on the symmetry axis of the three bottom spheres, as sketched in Fig. 1c. As this figure represents a cut through Fig. 1a along the dashed line, the two bottom spheres to the left of Fig. 1a are not visible in Fig. 1c. The liquid surface is represented by the circular dotted curve with radius r, as it just touches the top sphere.

Note that the assumption of a spherical liquid surface is an approximation, since it ignores interactions with neighboring pores. The shape of the liquid surface outside of the tetrahedral nook will influence its precise position relative to the surface of the top sphere. However, it is to be expected that this effect is small, since the liquid bulge invading the pore is connected to the liquid interface in neighboring pores only through the rather narrow throat regions.

It is now a matter of elementary geometry to write down a condition for the spherical liquid surface to touch the top sphere in a single point. With the help of Fig. 1d we immediately see by means of the cosine theorem that

$$y^2 = r^2 + R^2 - 2rR\cos\theta \tag{1}$$

Note that the contact angle appears at this place in the sketch because each of its two arms makes a right angle with one of the surfaces in contact. It is furthermore easy to see that $x^2 = 4R^2/3$, due to the geometry of the tetrahedral arrangement of the spheres.

From eq. (1), we readily find

$$\frac{r}{R} = \cos\theta + \sqrt{\left(\frac{y}{R}\right)^2 - \sin^2\theta}. \tag{2}$$

*Electronic address: r.seemann@physik.uni-saarland.de

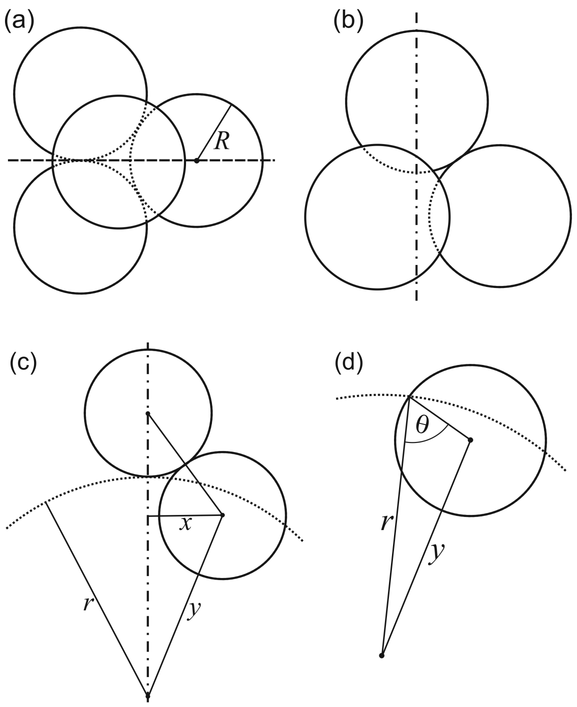

FIG. 1: Top: Sketch of a tetrahedral arrangement of spheres, as it frequently appears in random piles of spherical particles. (a) Top view. The dashed line indicates the cut used for Fig. 1a. (b) Side view. The dash-doted line indicates the symmetry axis of the tetrahedron. The lower left circle represents two spheres one of which eclipses the other. Bottom: The liquid front invades from below (c) The geometry of the tetrahedral arrangement of spheres (solid curves), and of the liquid interface within (dotted curve). (d) Construction for determining the relation between the auxiliary variable y and the contact angle, θ.

To characterize the maximal Laplace pressure of a liquid meniscus in such a pore we have to infer its minimal radius of curvature. Since $r(y)$ is strictly monotonous, r has a minimum if y does. This is the case if the center of the spherical liquid interface lies within the plane spanned by the centers of the three spheres in contact forming the throat. The corresponding minimum value of y is given by

$$y_{min} = \frac{2R}{\sqrt{3}}, \tag{3}$$

i.e., $y_{min} = x$. Inserting this in eq. (2) yields

$$\frac{r_{min}}{R} = \cos\theta + \sqrt{\frac{1}{3} + \cos^2\theta}. \tag{4}$$

Resolving for $\cos\theta$ yields

$$\cos\theta = \frac{Q}{2} - \frac{1}{6Q}, \tag{5}$$

with $Q = r_{min}/R$.

The distance H of the center of the top sphere from the plane spanned by the centers of the three bottom spheres is

$$H = \sqrt{(2R)^2 - (\frac{2}{3}R\sqrt{3})^2} = \sqrt{\frac{8}{3}}R. \tag{6}$$

Inserting then $r_{min} = H - R$ into eq. (5) leads to

$$\cos\theta = 0.0532, \tag{7}$$

and hence $\theta \approx 87°$. Consequently, for contact angles smaller than 87 degrees, there can be no HJ in this geometry because they are precluded by the liquid bulge touching the opposite wall of the pore before p_L decreases.

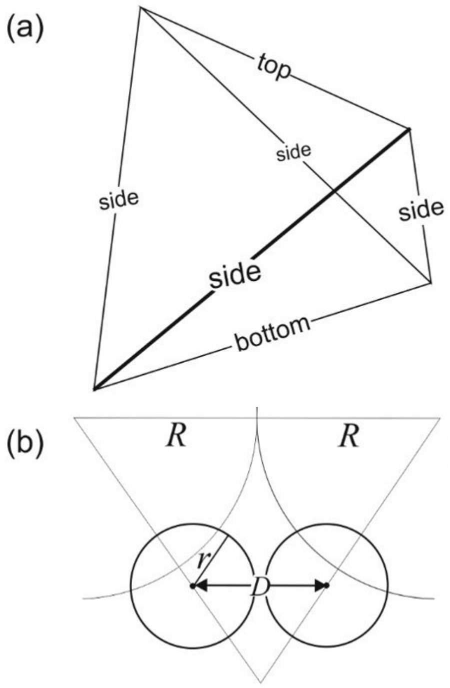

FIG. 2: (a) Here the tetrahedron is placed with one edge to the bottom. The front is imagined to invade from below, thus intruding through the two side faces sharing the bottom edge. (b) Cross section of top figure, normal to the direction of the bottom edge. Geometry of tetrahedral environment with two spherical liquid menisci invading a pore through two neighboring throats.

Two liquid bulges invading

Now let us assume that the tetrahedral arrangement of spheres is oriented as sketched in Fig. 2a, with one edge placed 'horizontally' on the bottom, in plane with the invading front. The liquid then invades the pore through two opposing throats, with two liquid interfaces approaching each other. It should be noted that since we are considering the quasi-static regime, the Laplace pressure must be spatially constant along the invading front, hence the radii of the two liquid bulges must be equal.

The condition of the transition is that the two liquid spheres just get into contact when r is minimal, $r = r_{min}$. From Fig. 2 we can directly deduce by means of the intercept theorem that

$$\frac{R/\sqrt{3}}{R\sqrt{3}} = \frac{D}{2R}, \tag{8}$$

and hence $D = 2R/3$. The spheres are touching each other when $D = 2r_{min}$, or when $Q = 1/3$. Inserting this condition into eq. (5), we obtain the condition for the transition to the Haynes Jumps (HJ) regime as

$$\cos\theta = -\frac{1}{3}, \tag{9}$$

or $\theta = 109.5°$.

Three liquid bulges entering

The orientation of the pore relative to the liquid front may be such that three throats are invaded at the same time. In this case we may imagine that the tetrahedral arrangement of Fig. 1 is turned upside down, with only one sphere at the bottom, and the liquid front invading from below. We have already mentioned that the size of these

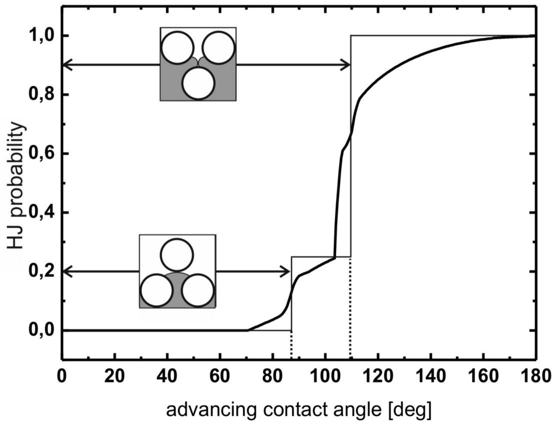

FIG. 3: The probability that a pore is being filled by HJ, as a function of the advancing contact angle of the invading phase.

bulges must be all the same, because they must all have the same Laplace pressure. Hence the condition for either of the three bulges coalescing is identical to the condition of coalescence for two invading bulges. Hence for a pile of perfectly mono-disperse spherical beads in mutual contact, we expect HJ to appear first at a contact angle of 87 degrees, and to become completely dominant if the contact angle exceeds 109.5 degrees (cf. Fig. 3).

Statistical weights

What is left to do is to add up the probabilities of HJ to occur, while observing the correct statistical weights. We assume that the pore under consideration is located at the advancing front. This can be expressed by assuming that at least one of the four throats entering the pore connects to an empty neighboring pore, and at least one throat leads to an already filled neighboring pore. This said, there are only two more throats to consider, which we call the *contingent* neighboring pores. If we further assume that the probability that a neighboring pore is filled is one half (as we are sitting right at the front), we can immediately calculate the statistical weights of the different configurations.

Configurations in which only one neighboring pore is filled correspond to those where only one bulge enters the pore under consideration. In this case, both of the contingent neighboring pores are empty. Hence the statistical weight of this process is $(\frac{1}{2})^2 = \frac{1}{4}$.

Configurations which have three neighboring pores already filled correspond to both contingent pores filled, hence their statistical weight is $\frac{1}{4}$ as well. These are the cases in which three bulges enter the pore under consideration at the same time.

Configurations which have just two filled neighboring pores are then to be considered with a statistical weight of $\frac{1}{2}$. In these cases, we have two bulges entering simultaneously.

As a result, we expect that as we gradually increase the contact angle, HJ will first occur at $87°$ with a statistical weight of 1/4 and become dominant at $109.5°$ (solid stepped line in Fig. 3). The step heights in Fig. 3 corresponds to the statistical weights.

Non-ideal pores

Next we are interested how this result changes if the tetrahedron is not perfectly closed, as it is the case for many pores in a random pile of spheres. Fig. 4 shows the distribution of the angles between neighboring contact points on the spheres in a random dense pile, as determined by means of x-ray microtomography [1]. In a perfect fcc or hcp piling, there would be only sharp peaks at 60 and 120 degrees from the tetrahedral environments and a peak at 90 degrees from the octahedral environments. The latter is completely absent here indicating the random nature of the pile. The more dominant is the peak at 60 degrees (and its 'mirror image' at 120 degrees) due to the strong preference for tetrahedral arrangments in random close packing.

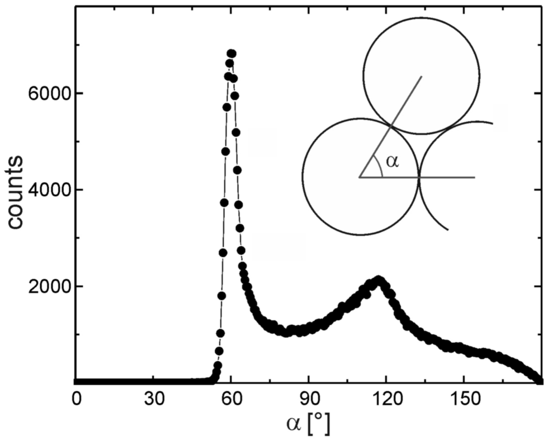

FIG. 4: The distribution of mutual angles between spheres in a random packing, as determined experimentally by x-ray microtomography [1].

The dominance of the peak at 60 degrees indicates that only relatively few contacts will be 'open', i.e., will have some finite gap between the surfaces of adjacent spheres. In what follows, we therefore consider tetrahedral arrangments which have only one of their contacts open, assuming that arrangements with two (or more) open contacts are sufficiently sparse for being safely neglected here. We will find that the effect of the wide distribution of angles α is merely to smear out the transition with respect to the result obtained for ideal tetrahedral pores, but not to shift its position or changing its qualitative appearance. Hence it appears indeed very unlikely that arrangements with more than one open contact would have any important effect.

Single liquid bulge invading

As obvious from Fig. 1a, there are three contacts between the spheres at the bottom and three contacts involving the top sphere. Opening these contacts has different effects on the transition, so they must be treated separately.

Open contact between bottom spheres

In order to have the same contact angle θ with all three bottom spheres, the spherical bulge invading from below must have its center on the dash-dotted line in Fig. 1c if all spheres are in contact. If the bottom spheres are not all in contact, this line comes to lie on the center of the circumcircle of the triangle formed by the three centers of the bottom spheres. The radius of this circumcircle, ϱ, then takes the role of the distance x we had in Fig. 1 and in the corresponding formulas. One finds

$$\varrho = R\sqrt{\frac{2}{1+\cos\alpha}} \tag{10}$$

where α is the angle opposing the open contact (cf. Fig. 4). Inserting ϱ for y in eq. (2) leads to

$$\cos\theta = \frac{Q}{2} - \frac{1-\cos\alpha}{2Q(1+\cos\alpha)} \tag{11}$$

instead of eq. (5).

If $\alpha \neq 60°$, the centers of the fluid bulge and the top sphere are not exactly aligned vertically. This deviation is small, however, and enters only to second order into the critical distance of the centers of the spheres. It is therefore neglected in all forthcoming considerations, since already the representation of the fluid interface by a sphere is only approximate.

The distance H of the center of the top sphere to the plane of the centers of the three bottom spheres is simply given by

$$H^2 = (2R)^2 - \varrho^2 \tag{12}$$

and must be equal to $R + r_{min}$ at the transition. We then obtain the liquid sphere minimum radius at which the liquid interface touches the top sphere as

$$Q := \frac{r_{min}}{R} = \sqrt{4 - \frac{2}{1+\cos\alpha}} - 1 \tag{13}$$

and hence the transition by inserting this into eq. (11). This gives rise to a shift of the critical angle for HJ to occur towards larger contact angles.

Open contact at top sphere

When the open contact is at the top sphere, the distance of the center of the top sphere from the plane spanned by the centers of the three bottom spheres is given by

$$H(\alpha) = R\sqrt{\frac{8}{3}}\sqrt{1+\cos\alpha - 2\cos^2\alpha}, \tag{14}$$

as can be shown by means of elementary geometry. Hence for Q we have

$$Q = \sqrt{\frac{8}{3}}\sqrt{1+\cos\alpha - 2\cos^2\alpha} - 1, \tag{15}$$

but eq. (5) remains valid. As a result, we find that this leads to a shift of the transition towards smaller contact angles.

Using the cumulative angle distribution from Fig. 4, we can now calculate for any advancing contact angle θ in what fraction of pores we expect HJ to happen. If there is no open contact, we have seen that for $\theta > 87°$ a HJ is sure to happen, but none below. Hence for all perfect tetrahedra, the resulting curve would be zero for angles below 87 degrees, where it jumps discontinuously to one, and remains there for all larger angles.

We have seen that if there is an open contact between two of the bottom spheres where the front is assumed to invade, the angle required for a HJ will be larger. In contrast, if the open contact is at the top sphere, a HJ is easier to achieve, and it will happen already at lower contact angles. Hence the open contacts give rise to two wings of the sought cumulative distribution. One wing extending from 87 degrees to larger angles, which is due to the open contacts within the invading front (i.e., between bottom spheres), and one wing extending from 87 degrees to smaller angles, which is due to the open contacts opposite to the invading front (i.e., at the top sphere). The smoothing effect is shown in Fig. 3.

Two liquid bulges invading

If two neighboring pores are already filled, two bulges of equal pressure (hence of equal radius) will invade the pore under consideration through two neighboring throats. These bulges will meet before they meet the opposite pore wall. This can be judged by the fact that the critical angle for HJ for two neighboring liquid bulges entering an ideal pore is 109.5°, well above the 87 degrees derived for HJ against the opposite pore wall. Hence the reunion of neighboring liquid bulges is more difficult to avoid, i.e., requires higher contact angles at the same Laplace pressure.

Top edge open

If we imagine again the tetrahedron to be placed with one edge (straight connection between the centers of two of its spherical grains) on the bottom and the opposite edge to the top, as sketched in Fig. 2a, we may investigate what results of the contact between the two top spheres (marked ‘top’ in the figure) is not perfect. Using again the angle

α occurring in Fig. 4, we find for the length of the top edge the expression $2R\sqrt{2-2\cos\alpha}$. The intercept theorem (eq. (8)) then yields

$$D = \frac{2R\sqrt{2-2\cos\alpha}}{3}. \tag{16}$$

Setting $2r_{min} = D$ as above, we have

$$Q = \frac{\sqrt{2-2\cos\alpha}}{3}, \tag{17}$$

which we can again insert in eq. (5) to obtain the advancing contact angle below which HJ cannot occur, for given angle α. Using the distribution of α, we can then calculate the probabilities of HJ as a function of θ. The result is that the transition becomes smeared out towards smaller contact angles.

Bottom edge open

The open contact may as well (with the same probability) be at the bottom edge (marked 'bottom' in Fig. 2a). The radius r of the invading bulges at maximum Laplace pressure is then given by eq. (11). At the same time, we still have $2r_{min} = D$, or $Q = 1/3$. This leads to

$$\cos\theta = \frac{2\cos\alpha - 1}{3\cos\alpha + 3}, \tag{18}$$

with the effect of smearing the transition towards larger angles.

Side edge open

If one of the side edges is open, we realize that the two liquid bulges entering the pore are doing so through throats with different geometry. We must remember that the Laplace pressure is assumed to be constant throughout the invading front in the quasistatic picture. Consequently, when the liquid bulge entering the throat with the open edge has its minimum radius of curvature, the radius of the other bulge must be the same. But since the other throat is smaller, this bulge will then not yet have reached the narrowest point of the throat. The center of the sphere which represents its foremost shape is then not in the plane spanned by the three spheres forming the throat, but lies outside by a distance h from that plane. It is elementary to show that

$$\frac{h}{R} = \sqrt{\frac{1-\cos\alpha}{1+\cos\alpha} - \frac{1}{3}}. \tag{19}$$

Projection onto the line connecting the two centers of the liquid bulges yields a factor of $\cos 35.26^\circ = 0.816$ to be applied to h. Following the discussion leading to eq. (9), we find the condition

$$2Q = \frac{2}{3} + 0.816\frac{h}{R} = \frac{2}{3} + 0.816\sqrt{\frac{1-\cos\alpha}{1+\cos\alpha} - \frac{1}{3}}. \tag{20}$$

Inserting then Q in eq. (11), we obtain the condition on the contact angle for HJ. As the gap between the two spheres forming the throat opens up wider, the opposing liquid bulge becomes less and less important for precluding a HJ. The intruding bulge will then rather touch the opposite pore wall, which has been discussed before and the transition is smeared towards larger contact angle.

Three liquid bulges invading

Here we imagine again that the tetrahedral arrangement of Fig. 1 is turned upside down, with only one sphere at the bottom, and the liquid front invading from below. In this case there are three possibilities that the open contact affects only one of the invaded throats, and three possibilities that the open contact is between two of the invaded throats. The latter case is identical to the case of two invaded throats with one side edge open. This has been discussed above. The other case involves two throats affected by the open contact. This is identical to the case of two invaded throats with the bottom edge open. This has been discussed as well further above. Hence within the statistical weight of 1/4 of three bulges invading at the same time, there are equally weighted contributions equivalent to the two-bulges case with open side edge, and the two-bulges case with open bottom edge, respectively.

Putting it all together

What remains is to put all contributions to the probability of HJ occurring together. We assume that the relative orientation of the pore under consideration with respect to the invading front is arbitrary and evenly distributed. This is justified if there is no texture or crystalline coordination in the pile, which we henceforth assume. Furthermore, we assume that the configurations of the individual pores can be described sufficiently well by assuming that there is only one open bead contact, and that the corresponding angle is distributed according to Fig. 4. We then can compute the total probability of HJ by adding up all contributions we have calculated above with the corresponding statistical weights. The result is the solid curve in Fig. 3. Clearly, the effect of the random nature of the pile, which renders the great majority of the pores non-ideal, is just to smear out the result obtained for the ideal pore, without any substantial shift occurring. This curve is used in the main article in comparison to the residual saturation with defending liquid.

---

[1] M. Scheel et al., *Nature Mat.* **7** (2008) 189.

Description movies

Video S1 Basalt bead pack

Movie shows cross sections through time resolved x-ray tomography images recorded while an aqueous ZnI_2-solution is flushed into an initially dodecane filled basalt bead matrix (sieve fraction (355 - 425)μm) with a contact angle of the aqueous phase of about 75 deg. Average front velocity is 3 $\mu m/s$, the cylindrical sample container has a diameter of 8 mm; the height of the imaged area is also 8 mm. According to the different x-ray absorption, the beads are displayed as white, the aqueous phase appears as grey and the oily phase as black. Time delays between individual tomograms are increased during the water flush. The total duration of the movie correspond to $\approx$200 min, respectively seven injected pore volumes.

Video S2 Glass bead pack

Movie shows cross sections through time resolved x-ray tomography images recorded while an aqueous ZnI_2-solution is flushed into an initially dodecane filled glass bead matrix (sieve fraction (355 - 425)μm) with a contact angle of the aqueous phase of about 125 deg. Average front velocity is 3 $\mu m/s$, the cylindrical sample container has a diameter of 8 mm; the height of the imaged area is also 8 mm. According to the different x-ray absorption, the beads are displayed as grey, the aqueous phase appears as white and the oily phase as black. Time delays between individual tomograms are increased during the water flush. The total duration of the movie correspond to $\approx$300 min, respectively ten injected pore volumes.

Video S3 (animated GIF) Fast-tomos

Animated 3d representations of time resolved x-ray tomography images recorded while an aqueous ZnI_2-solution is flushed into an initially dodecane filled glass bead matrix (sieve fraction (355 - 425)μm, not shown in the images) with a contact angle of the aqueous phase of about 125 deg. Average front velocity is 3 $\mu m/s$, the cylindrical sample container has a diameter of 8 mm. Time delays between consecutive tomograms is 5.7 s.
