# Supplementary figures and images for: The Role of Local Instabilities in Fluid Invasion into Permeable Media
Source: Sci Rep. 2017 Mar 27;7:444. doi: 10.1038/s41598-017-00191-y (PMC5427855; doi:10.1038/s41598-017-00191-y)

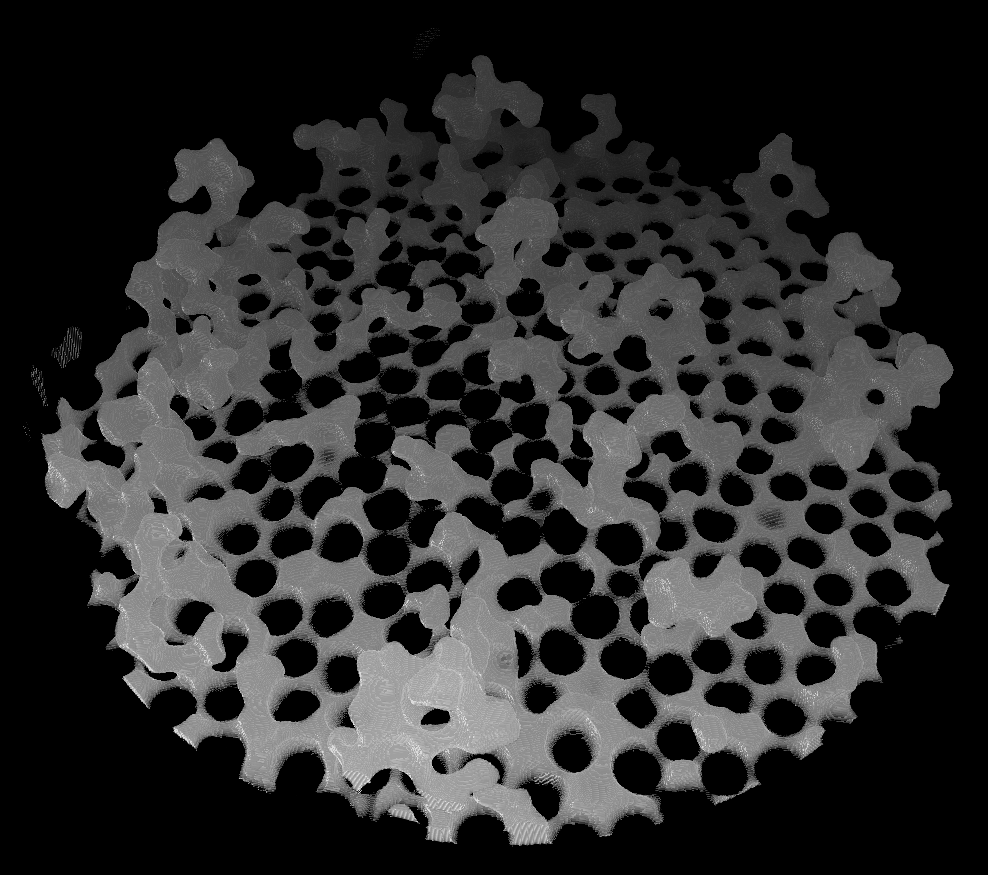

Supplement: Supplementary file 4 — S3 Fast-tomos [file 41598_2017_191_MOESM4_ESM.gif]
